# The NAVIGATE Registry: Early Endovascular Repair of the Ascending Aorta With the Nexus Ascending Module

**DOI:** 10.1093/icvts/ivag128

**Published:** 2026-04-30

**Authors:** Víctor X Mosquera, Andrea Ferreiro, Said Abisi, Luis Riera, Alexander Maßmann, Arun Pherwani, José Garrido

**Affiliations:** Servicio de Cirugía Cardíaca, Complejo Hospitalario Universitario de A Coruña, A Coruña, Newcastle 15006, Spain; Hospital Universitario Virgen de las Nieves, Granada 18014, Spain; Guy’s and St. Thomas’ NHS Foundation Trust, London SE1 7EH, United Kingdom; Hospital Universitario La Paz, Madrid 28046, Spain; Robert-Bosch-Krankenhaus, Stuttgart 70184, Germany; Keele University School of Medicine, University Hospitals of North Midlands NHS Trust, Newcastle ST5 5BG, United Kingdom; Hospital Universitario Virgen de las Nieves, Granada 18014, Spain

**Keywords:** ascending aorta, endovascular repair, Nexus endograft, pseudoaneurysms, type A aortic dissection

## Abstract

**Objectives:**

Open repair remains the standard for ascending aortic pathology but carries high morbidity in frail or inoperable patients. This study evaluates the feasibility and early outcomes of endovascular repair using the Nexus Ascending Module, a CE-marked device designed for zone 0.

**Methods:**

NAVIGATE is a retrospective multicentre European registry including 8 high-risk patients treated between 2023 and 2025 across 6 tertiary centres. All had focal zone 0 pathology and were deemed unsuitable for open repair. Procedures were planned with ECG-gated CT angiography and performed transfemorally. Technical success, perioperative complications, 30-day mortality, and follow-up outcomes were assessed.

**Results:**

Median age was 80.0 years (IQR: 59.5-80.5), and 87.5% had prior cardiac surgery. The median EuroSCORE II was 24.0% (IQR: 19.7%-44.0%). Pathologies included contained rupture/pseudoaneurysm (*n* = 4), mycotic pseudoaneurysm (*n* = 3), and type A DeBakey II dissection (*n* = 1). Technical success was achieved in all patients; 2 required immediate overlapping stent-grafts for complete sealing. No stroke, valve dysfunction, coronary compromise, tamponade, type I/III endoleak, conversion to surgery, or in-hospital/30-day mortality occurred. One access-related complication was observed.

Median ICU and hospital stays were 1 and 5 days. At a median follow-up of 15 months (IQR: 12-24), no aortic reinterventions or device-related complications occurred. Two patients died from respiratory disease (12 months) and persistent infection (4 months).

**Conclusions:**

This small multicentre case series suggests that endovascular repair of isolated zone 0 ascending pathology using the Nexus Ascending Module is technically feasible in highly selected patients; however, these findings are preliminary and hypothesis-generating.

## Graphical abstract

**Figure:**
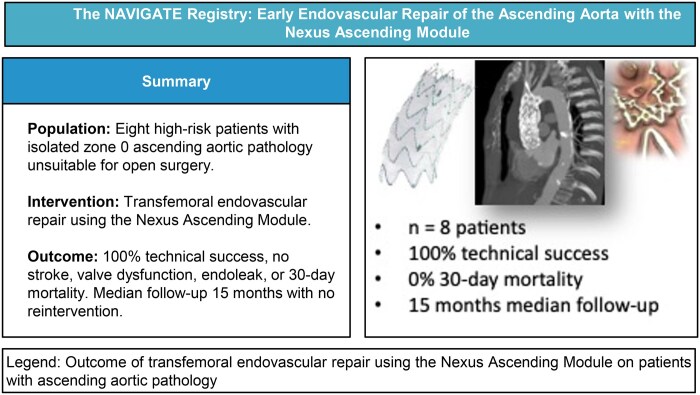

## INTRODUCTION

Open surgical repair remains the gold standard for ascending aortic pathology, providing durable outcomes but with significant morbidity and mortality, particularly in elderly or high-risk patients. Early mortality ranges from 5%-15% in emergent or complex cases, largely due to neurological and cardiac complications^1–4^, and up to 20% of patients may be considered inoperable because of frailty or hostile anatomy^5^^,^^6^.

Although thoracic endovascular aortic repair (TEVAR) is established for descending disease, application in the ascending aorta remains challenging due to short landing zones, proximity to the valve and coronary ostia, large vessel diameters, and high pulsatility^6–9^. Dedicated devices are therefore required. Focal non-dissecting lesions—such as pseudoaneurysms, penetrating ulcers, and saccular aneurysms—may be particularly suitable for endovascular repair when the root and arch are preserved^10^^,^^11^.

Early studies using ECG-gated imaging and dedicated devices have reported technical success rates exceeding 95%^6^^,^^9^, although concerns remain regarding valve interference, coronary compromise, and stroke^5^^,^^8^.

In this context, the NAVIGATE registry reports one of the largest multicentre experiences of isolated zone 0 ascending repair using the CE-marked Nexus Ascending Module, evaluating the real-world performance of a standardized platform in high-risk non-surgical patients.

## PATIENTS AND METHODS

### Study design and ethical approval

This retrospective multicentre analysis of the NAVIGATE registry, an investigator-initiated European database, evaluates the feasibility, safety, and early outcomes of endovascular treatment of the ascending aorta using the Nexus Ascending Module (Endospan Ltd., Herzliya, Israel). The registry includes patients from 6 tertiary centres in Spain, Germany, and the United Kingdom. The study was approved by the Ethics Committee for Research with Medicines of Galicia (CEIm-G), Spain (approval 2025/248), and all patients provided written informed consent in accordance with the Declaration of Helsinki.

### Patient selection

Between January 2023 and June 2025, eight high-risk or inoperable patients with isolated ascending aortic pathology (zone 0) were enrolled. All cases were reviewed by multidisciplinary heart teams at each centre, including cardiac and vascular surgeons, anesthesiologists, and interventional radiologists, and open surgery was considered contraindicated or associated with prohibitive risk. Surgical risk assessment included EuroSCORE II, frailty, comorbidity burden, redo sternotomy risk, anatomical complexity, and systemic factors such as infection or severe pulmonary disease. Frailty was assessed clinically but not with a standardized scale due to the retrospective multicentre design. Treatment strategies were harmonized through a standardized device platform, ECG-gated CTA planning, and uniform outcome definitions.

Inclusion criteria were:

- Focal ascending aortic pathology restricted to zone 0 (between the sinotubular junction and the origin of the brachiocephalic trunk),
- Adequate proximal and distal landing zones for endograft deployment,
- Unsuitability for open surgical repair due to prohibitive comorbidity, frailty or challenging reoperations with high procedural risk.

Eligible pathologies included:

- Pseudoaneurysms
- Penetrating aortic ulcers
- Saccular aneurysms
- Focal ascending aortic dissection limited to the ascending segment (Stanford type A/DeBakey type II), with no extension into the aortic arch or descending thoracic aorta.

Exclusion criteria included:

- Extensive dissections involving the arch or descending thoracic aorta (DeBakey type I),
- Involvement of the aortic root or coronary ostia,
- Prior aortic root replacement with a valved conduit,
- Need for hybrid supra-aortic debranching or combined procedures.

### Procedural technique

All procedures were performed in hybrid operating rooms under general anaesthesia with systemic heparinization and rapid pacing. The Nexus Ascending Module was delivered transfemorally. This pre-curved, self-expanding nitinol stent graft is designed for ascending aortic anatomy and includes dedicated proximal and distal sealing zones with a reinforced proximal ring to enhance wall apposition and reduce migration risk.

Anatomical suitability was determined using ECG-gated CTA and required: proximal and distal landing zone length ≥15 mm; landing zone diameters within the device sizing matrix (approximately 28-40 mm); device oversizing between 10%-20%; coronary clearance ≥10-15 mm; absence of root involvement; and acceptable ascending aortic curvature permitting coaxial deployment.

Preoperative ECG-gated CTA was used for anatomical planning and device sizing. Procedures were guided by fluoroscopy, angiography, and intraoperative transoesophageal echocardiography (TEE) in all cases except patient no. 6. Completion angiography confirmed correct device positioning and absence of endoleaks.

### Postoperative management and follow-up

Patients were admitted to the intensive care unit (ICU) for routine monitoring. Postoperatively, patients were maintained on single antiplatelet therapy with aspirin (75-100 mg daily) unless contraindicated. Follow-up imaging with CTA was performed prior to discharge and at 30 days to assess device integrity and aneurysm exclusion. Clinical evaluation focused on neurological status, access-site complications, and systemic recovery.

### Outcomes

The primary end-point was technical success, defined as successful device deployment at the intended location with complete exclusion of the lesion, no type I or III endoleak, no unplanned adjunct procedures, and no conversion to open surgery.

Secondary end-points included:

- 30-day all-cause mortality
- Neurological complications (stroke, TIA)
- Cardiac tamponade
- Major bleeding (≥4 units transfused or reintervention)
- Access-related vascular complications
- Postoperative infections
- Length of ICU and hospital stay

### Statistical analysis

Given the limited sample size (*n* = 8), all analyses were descriptive. Continuous variables are presented as median and interquartile range (IQR), while categorical variables are reported as counts and percentages. No inferential statistical tests were applied. Data completeness was 100%. Statistical analyses were performed using R version 4.3.1 (R Foundation for Statistical Computing, Vienna, Austria). Statistical reporting adhered to contemporary recommendations for surgical cohort studies and small-sample registry data ^13^.

## RESULTS

**Figure 1.**
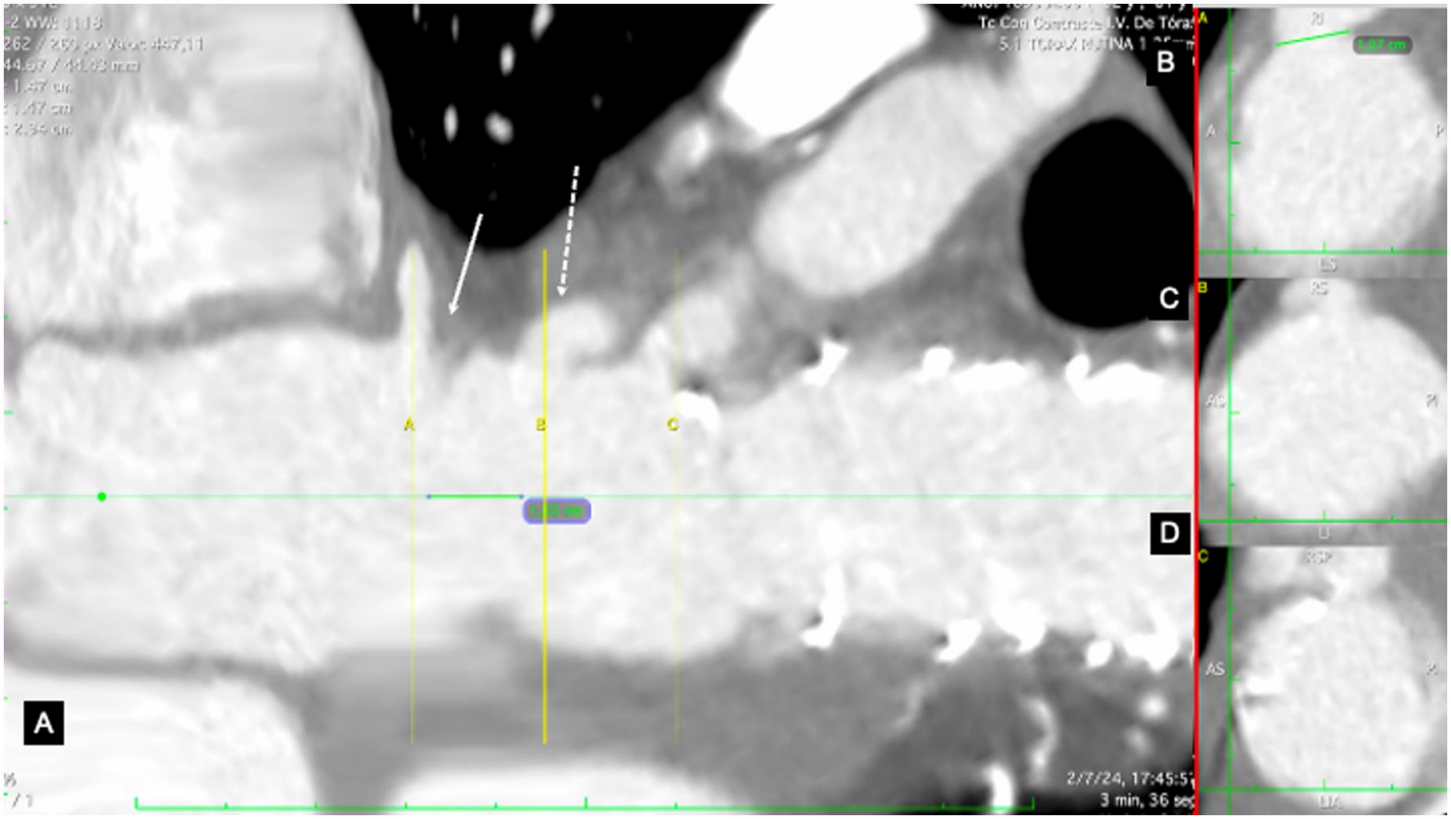
Imaging of Patient no. 2 with Prior Frozen Elephant Trunk. (A) CT Reconstruction Showing the Pseudoaneurysm at the Proximal Anastomosis and the Distance to the Translocated LCCA Bypass (Solid Arrow: entry Tear; Dashed Arrow: LCCA Bypass). (B–C) Alignment of the Entry Tear and LCCA Bypass. (D) Origin of the Translocated Brachiocephalic Trunk Bypass.

**Figure 2.**
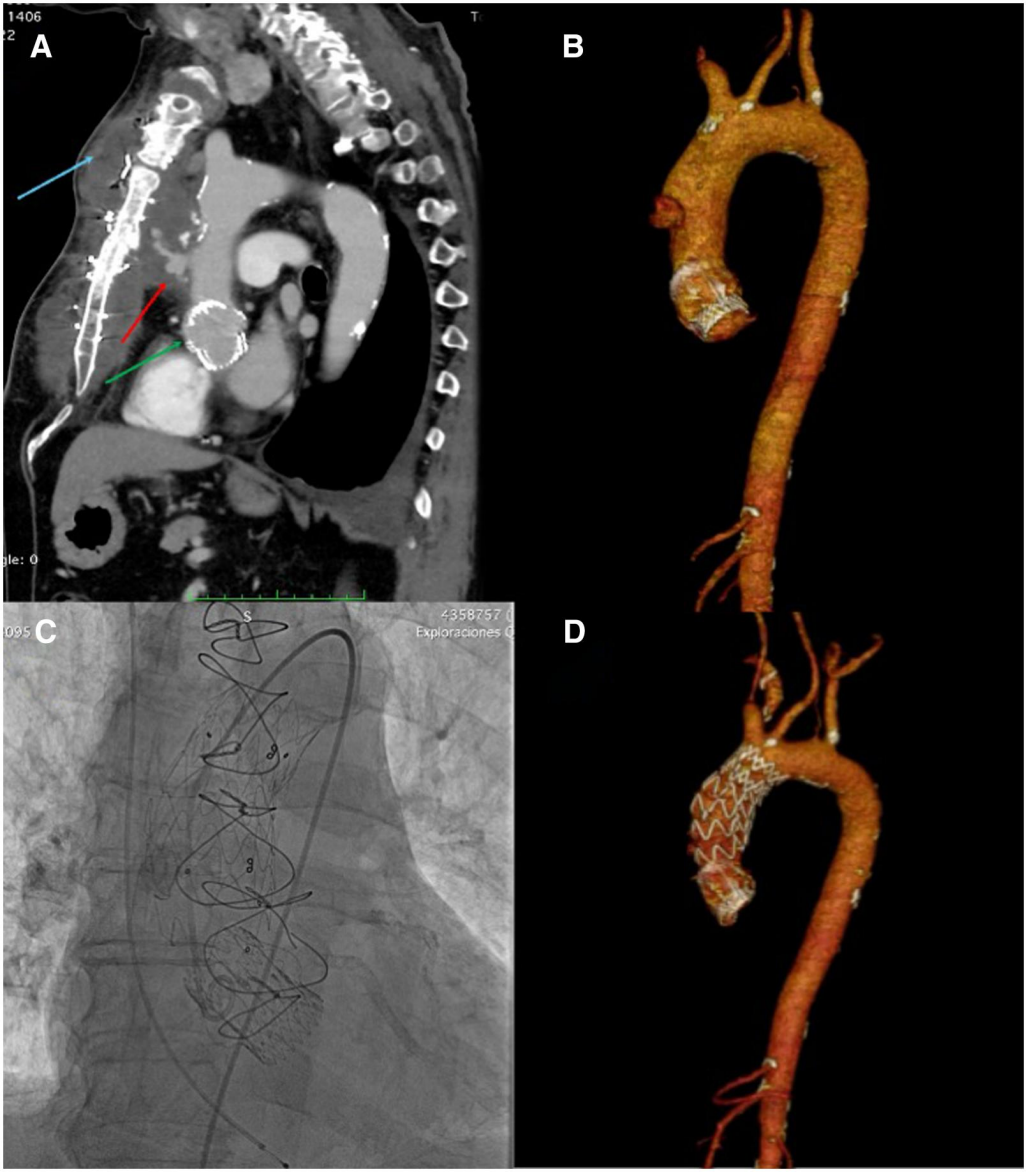
Preoperative, Intraoperative, and Postoperative Imaging of Patient no. 3. (A) CTA Showing Presternal Hematoma, Ascending Pseudoaneurysm, and Perceval Valve. (B) 3D CT Reconstruction. (C) Fluoroscopy during Transfemoral Nexus Ascending Module Deployment. (D) Postoperative CTA Confirming Exclusion without Endoleak.

Between January 2023 and June 2025, 8 patients were treated with the Nexus Ascending Module as part of the NAVIGATE registry. All cases presented with focal pathology confined to zone 0 of the ascending thoracic aorta, and all were deemed at prohibitive surgical risk. The median age was 80.0 years (IQR: 59.5-80.5), and 62.5% were female. The entire cohort exhibited a significant burden of comorbidities, including hypertension (6 pts, 75%), frailty (6 pts, 75%), chronic obstructive pulmonary disease (5 pts, 62.5%), chronic kidney disease (4 pts, 50%), and coronary artery disease (4 pts, 50%). Previous cardiac surgery was present in 7 out of 8 patients (87.5%) (Table 1). These included 2 prior frozen elephant trunk (FET) procedures (Figure 1; Video 1), one replacement of the ascending aorta, one aortic valve replacement with a sutureless bioprosthesis (Figure 2; Video 2), one surgical pericardial drainage due to recurrent tamponade, one coronary artery bypass grafting (CABG), one extended thymic tumour resection with vascular reconstruction and one surgical pericardial drainage performed via median sternotomy. Only one patient had no history of prior cardiac intervention.

**Table 1.** Baseline Clinical and Demographic Characteristics of the Study Cohort

Patient	Sex	Age (years)	HTN	COPD	CAD	PAD	Frailty	CKD	Logistic EuroSCORE II (%)	Previous cardiac surgery	Indication
**1**	F	51	Yes	No	No	No	No	No	26,68%	Yes (FET)	Pseudoaneurysm of the proximal FET anastomosis
**2**	F	61	Yes	No	No	No	Yes	No	11%	Yes (FET)	Pseudoaneurysm of the proximal FET anastomosis
**3**	M	83	Yes	Yes	Yes	No	Yes	Yes	19,70%	Yes (AVR)	Pseudoaneurysm arising from an ascending aortic conduit
**4**	M	80	Yes	Yes	Yes	No	Yes	No	24%	No	Type A aortic dissection (DeBakey II)
**5**	F	80	Yes	No	No	No	Yes	Yes	21,94%	Yes (pericardial fluid drainage)	Ascending aortic mycotic pseudoaneurysm
**6**	F	81	Yes	Yes	Yes	Yes	Yes	Yes	56,04%	Yes (CABG)	Ascending aortic mycotic pseudoaneurysm
**7**	F	58	No	Yes	No	No	Yes	No	44%	Yes (resection of thymic tumour)	Ascending aortic mycotic pseudoaneurysm
**8**	M	75	No	Yes	Yes	Yes	No	Yes	14,87%	Yes (previous ascending aortic replacement)	Pseudoaneurysm arising from an ascending aortic conduit

Abbreviations: AVR, aortic valve replacement; CAD, coronary artery disease; CKD, chronic kidney disease; COPD, chronic obstructive pulmonary disease; FE, frozen elephant trunk; HTN, hypertension; PAD, peripheral artery disease.

ECG-gated CTA confirmed anatomical feasibility in all cases. The mean aortic lesion size was 19.8 ± 4.1 mm. The proximal landing zone diameter averaged 32.9 ± 1.3 mm, with a sealing length of 29.1 ± 22.6 mm. The distal landing zone diameter was 30.3 ± 0.6 mm, and its sealing length was 27.9 ± 9.6 mm.

**Figure 3.**
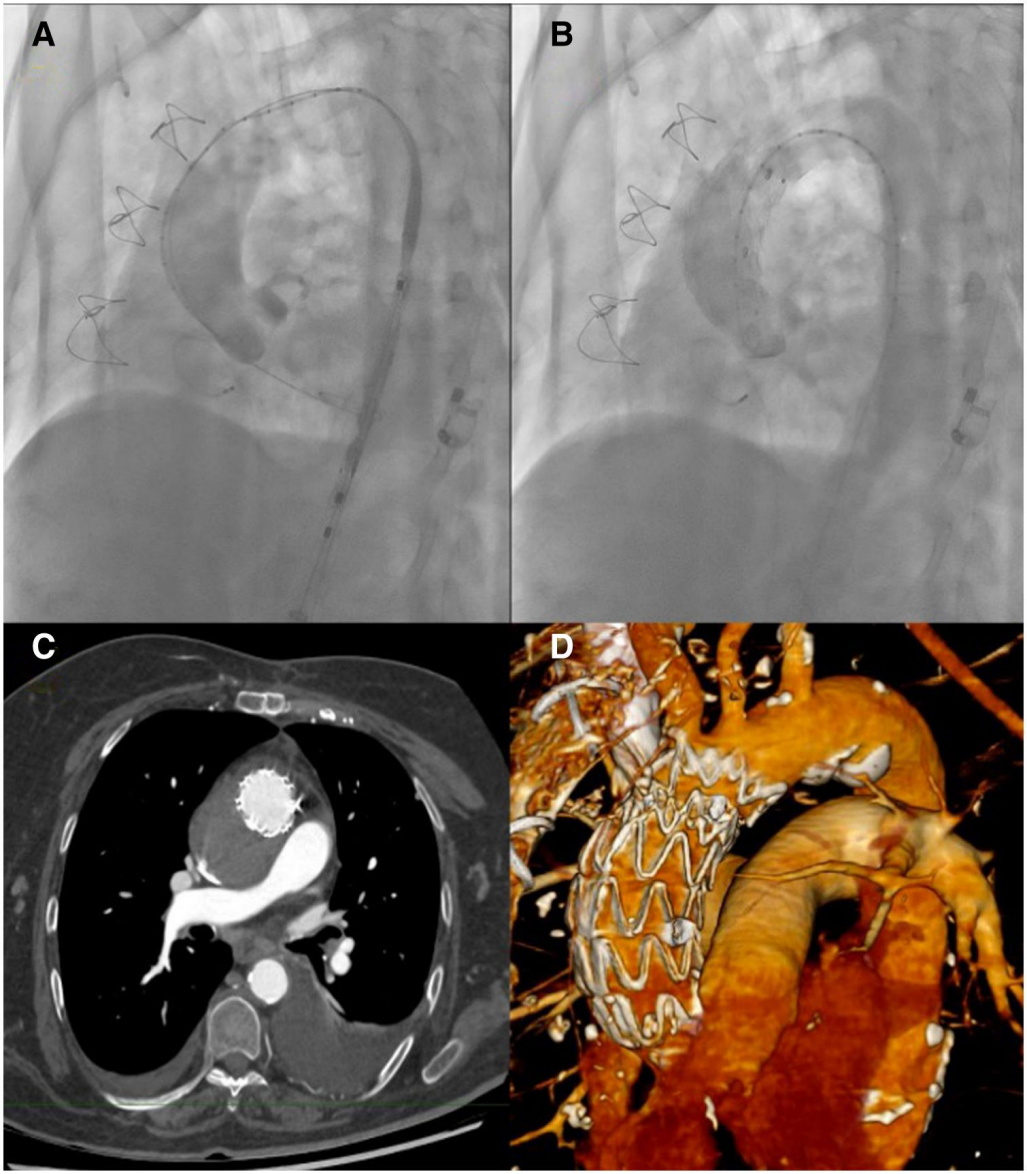
Imaging of Patient no. 5 with a Mycotic Ascending Pseudoaneurysm. (A) Pre-Deployment Angiography. (B) Completion Angiography after Nexus Ascending Module Implantation. (C) Postoperative CTA Confirming Correct Positioning and Preserved Valve and Coronary Flow. (D) 3D Reconstruction Showing Adequate Graft Apposition.

**Figure 4.**
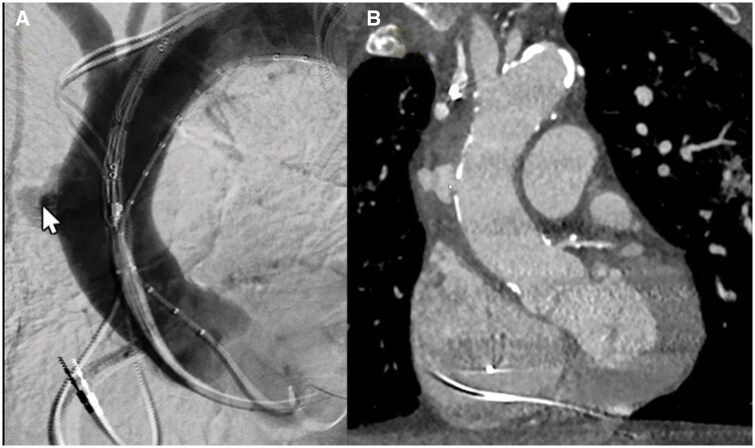
Imaging of Patient no. 6 with a Mycotic Pseudoaneurysm. (A) Intraoperative Angiography Showing the Lesion (Arrow). (B) Postoperative CTA Confirming Accurate Device Deployment and Exclusion. The Patient Died Four Months Later Due to Persistent Infection.

The underlying aortic pathology included 4 pseudoaneurysms (Figures 1 and 2), 3 mycotic pseudoaneurysms (Figures 3 and 4), and 1 limited ascending aortic dissection classified as Stanford type A/DeBakey type II, without extension beyond the ST junction.

Procedural data is shown in Table 2. Technical success was achieved in all patients. In this small cohort, no periprocedural stroke, transient ischaemic attack, aortic valve dysfunction, coronary compromise, cardiac tamponade, type I or III endoleak, or conversion to open surgery was observed. One access-related vascular complication occurred. There was no in-hospital or 30-day mortality. All procedures were performed via transfemoral access under general anaesthesia with rapid pacing. No additional endovascular maneuvers, arch stent-grafting, or conversion to open surgery was required.

**Table 2.** Procedural Details, Early and Late Clinical Outcomes of Patients Treated with the Nexus Ascending Module

Patient	Max. diameter and length (mm)	Proximal LZ diameter (mm)	Proximal LZ length (mm)	Distal LZ diameter (mm)	Distal LZ length (mm)	Nexus device (mm)	Number of stent-grafts per patient	Femoral access	Immediate outcome	Late outcome
**1**	60 × 120	38	20	33	30	40 × 40	1	Open	No complications	Alive, asymptomatic at 24-month follow-up
**2**	11.5 × 10.5	38	15	37	20	40 × 40	1	Open	No complications	Alive, asymptomatic at 15-month follow-up
**3**	33.9 × 13	31.8	13	34	50	40 × 70	1	Open	No complications	Alive, asymptomatic at 24-month follow-up
**4**	11.5 × 10.5	30	86	31.9	20	36 × 70	2 (36 × 40)	Open	No complications	Deceased at 12 months (respiratory disease)
**5**	30 × 40	30	18	30	24	36 × 70	1	Percutaneous	No complications	Alive, asymptomatic at 12-month follow-up
**6**	23 × 20	28	35	28	30	36 × 55	1	Percutaneous	Femoral pseudoaneurysm (closure device-related)	Deceased at 4 months (persistent infection)
**7**	25 × 30	36	30	33	20	40 × 55	1	Percutaneous	Bridge to open repair (homograft)	Alive after elective explant and homograft replacement
**8**	29.7 × 77	29.7	15	29.7	30	35 × 55	2 (36 × 70)	Percutaneous (converted to open due to bleeding)	Access site bleeding	Alive, asymptomatic at 2-month follow-up

Abbreviation: COPD, chronic obstructive pulmonary disease.

Regarding device selection, the most common graft lengths were 55 mm and 70 mm (*n* = 3 each), followed by 40 mm (*n* = 2). Graft diameters of 36 mm and 40 mm were each used in 4 patients. In 2 patients (nos 4 and 8), immediate additional overlapping stent-grafts were deployed intraoperatively to achieve complete proximal sealing. No arch stent-grafting or conversion to open surgery was required.

Patients were transferred to the ICU postoperatively for routine monitoring. The median ICU stay was 1.0 day (IQR: 1.0–2.0), and the median total postoperative hospital stay was 5.0 days (IQR: 4.0–8.25).

The early postoperative course was uneventful. No periprocedural stroke, cardiac tamponade, or aortic valve dysfunction occurred. Patient no. 6 developed a small femoral pseudoaneurysm related to vascular closure device failure in the setting of peripheral arterial disease. In patient no. 8, adjunctive shockwave angioplasty of the infrarenal aorta was required due to severe calcification, and femoral access was converted to surgical exposure because of bleeding. No postoperative infections, major bleeding, or thromboembolic complications were observed. Thirty-day mortality was zero.

The cohort’s median EuroSCORE II was 24.0% (IQR: 19.7%-44.0%), indicating high predicted surgical risk. Median follow-up was 15.0 months (IQR: 12.0-24.0) (Table 2). No type I endoleak, device migration, valve dysfunction, or reintervention occurred. Two patients died during follow-up: one from respiratory failure at 12 months (patient no. 4) and one from persistent infection at 4 months (patient no. 6). One patient (no. 7) underwent elective explant and homograft replacement after infection resolution without reinfection. The remaining 5 patients were alive, asymptomatic, and free of imaging evidence of endograft-related complications.

Two patients (nos. 1 and 2) with prior frozen elephant trunk developed proximal anastomotic pseudoaneurysms during hypertensive crises. Patient no. 2, a 61-year-old woman with CFS 7, COPD, diabetes, and prior DeBakey type I dissection, presented with a pseudoaneurysm at the proximal Thoraflex anastomosis (Figure 1; Video 1). Despite challenging anatomy (RCA—entry tear distance 16.4 mm; distal sealing zone 10.3 mm before the translocated LCCA bypass; LVOT angle 51°; curvature radius 29.7 mm), a 40 × 40 mm Nexus module achieved complete exclusion with preserved RCA and LCCA flow. The patient recovered uneventfully and was discharged on day 7, with durable sealing on follow-up CTA.

Patient no. 4 with severe pulmonary emphysema and cachexia underwent endovascular repair of a DeBakey type II dissection using a 36 × 70 mm Nexus module. An intraoperative type Ia endoleak was treated with an overlapping 36 × 40 mm proximal extension, achieving complete exclusion. The patient later died at 12 months from viral pneumonia.

Three patients (nos. 5-7) had mycotic pseudoaneurysms. Patient no. 5 (Figure 3) had *Escherichia coli* bacteremia from gangrenous cholecystitis and was treated with ceftriaxone followed by oral levofloxacin; PET-CT confirmed infection resolution and the patient remains alive and asymptomatic. Patient no. 6 (Figure 4; Video 3) had polymicrobial infection (*E. coli* and *Corynebacterium striatum*) treated with meropenem and cotrimoxazole but died four months later from persistent infection. Patient no. 7, with prior mediastinal radiotherapy and suspected infection without an identified pathogen, underwent endovascular repair as a bridge-to-surgery strategy, followed by elective explant and homograft replacement after antimicrobial therapy; the patient remains alive and neurologically intact.

## DISCUSSION

The management of ascending aortic pathology has traditionally relied on open surgery due to the proximity to the aortic valve, coronary ostia, and supra-aortic vessels, as well as the dynamic motion of the proximal aorta. However, endovascular repair has emerged as a potential alternative in selected patients deemed unfit for conventional surgery. The NAVIGATE registry represents one of the largest multicentre experiences of isolated zone 0 ascending aortic repair using a dedicated endograft (Nexus Ascending Module) without arch or descending extension.

Endovascular treatment of the ascending aorta remains technically challenging because of the short sealing zone between the sinotubular junction and brachiocephalic trunk, proximity to the aortic valve and coronaries, large vessel diameter, and high pulsatile stress^5^^,^^6^^,^^9^. These factors require dedicated stent-grafts specifically designed for this segment, while also raising concerns regarding migration or retrograde dissection. On the other hand, ascending endografting carries theoretical risk of retrograde or stent-induced dissection, particularly at the proximal landing zone. Although no such events were observed in this small cohort, long-term surveillance is mandatory.

Early studies suggest that focal, non-dissecting lesions may be suitable for endovascular repair. CT-based analyses indicate that up to 52% of ascending aneurysms or pseudoaneurysms may be anatomically feasible for endovascular treatment^5^. Localized lesions such as pseudoaneurysms, penetrating ulcers, and saccular aneurysms appear particularly suitable because of their confined morphology and relative sparing of the aortic root and arch^10–12^.

In the NAVIGATE cohort, all patients underwent transfemoral implantation of a single-module Nexus endograft. Despite a high-risk profile (median EuroSCORE II 24%) and frequent prior cardiac surgery, technical success was achieved in all patients, with no 30-day mortality, stroke, tamponade, or type I endoleak. Two cases required an additional overlapping stent graft for complete sealing, resulting in 10 devices implanted across 8 patients. One patient later required homograft replacement due to infection. These results compare favourably with earlier series using modified or off-label devices^10–14^.

**Figure 5.**
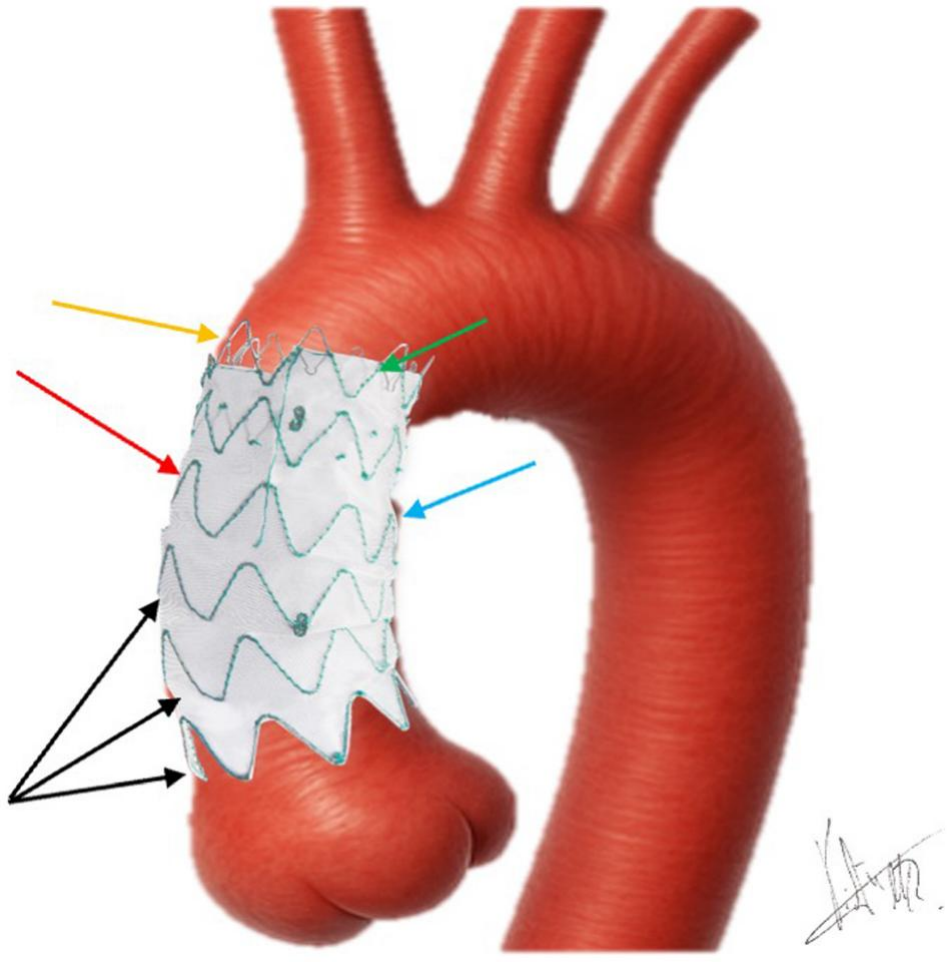
Design Features of the Nexus Ascending Module. The Device Adapts to the Asymmetric Geometry of the Ascending Aorta. Red Arrow: Anti-Buckling Spine. Yellow Arrow: scalloped Proximal Edge. Green Arrow: M-Shaped Struts Preserving Supra-Aortic Ostia. Blue Arrow: inner Curvature Springs. Black Arrow: inwardly Bent Strut Apices.

The Nexus design, incorporating a pre-curved configuration, anti-buckling spine, and dedicated sealing zones (Figure 5), addresses several anatomical challenges of the ascending aorta while avoiding arch manipulation or supra-aortic debranching. In this series, the approach proved particularly suitable for focal lesions such as pseudoaneurysms, penetrating ulcers, and saccular aneurysms, especially in patients with previous cardiac or aortic surgery.

**Video 1.**
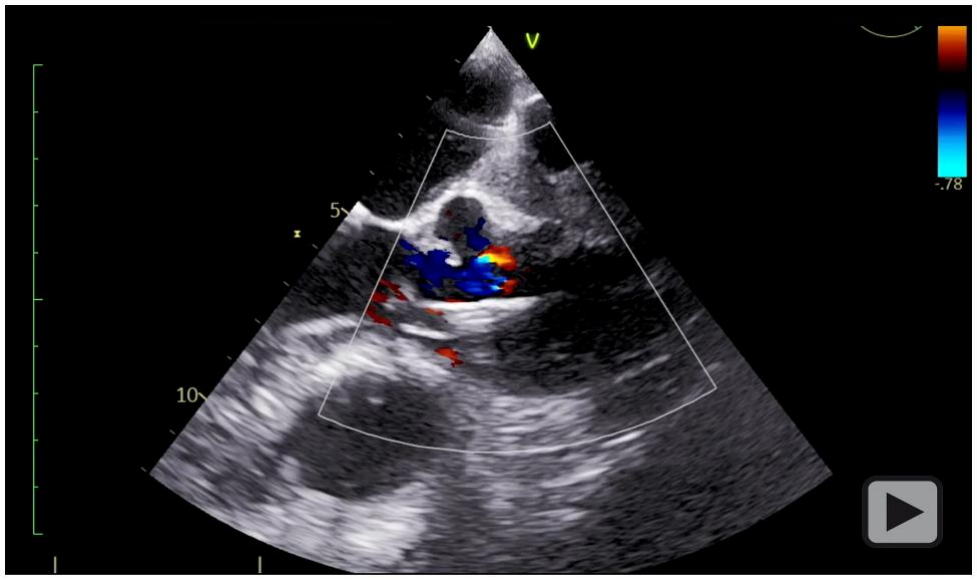
Fluoroscopy and TEE of Patient no. 2 during Nexus Ascending Module Deployment, Demonstrating Complete Exclusion without Compromise of the Aortic Valve, Coronary Ostia, RCA Flow, or the Translocated LCCA Bypass.

**Video 2.**
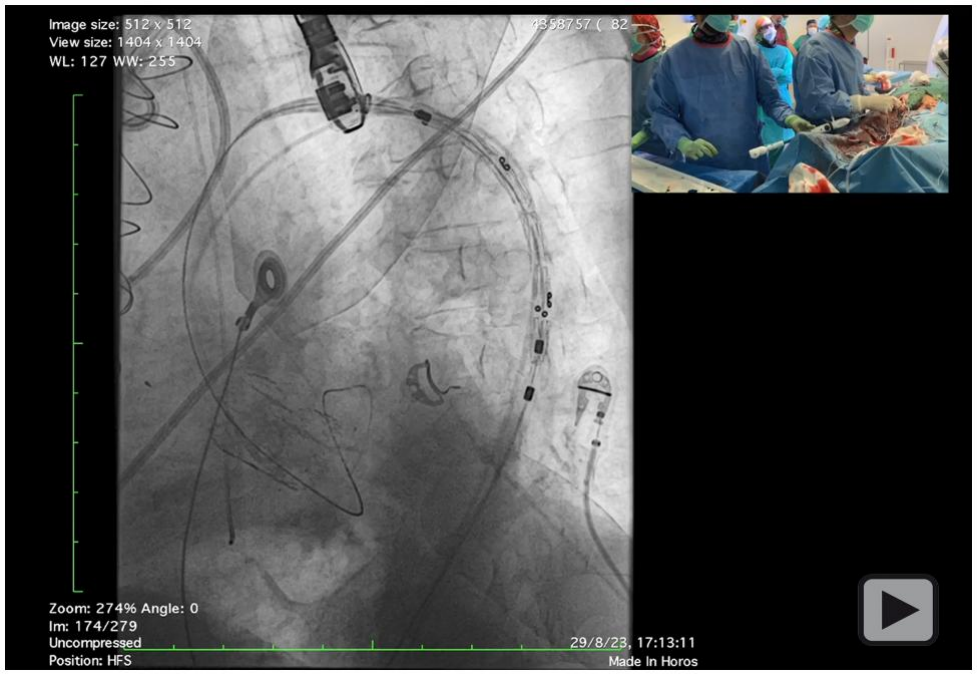
Endovascular Repair of a Ruptured Ascending Pseudoaneurysm in Patient no. 3. Fluoroscopy Shows Transfemoral Delivery and Deployment of the Nexus Ascending Module under Rapid Pacing. Completion Angiography Confirms Correct Positioning and Exclusion without Endoleak. The Perceval Valve Remained Functional.

**Video 3.**
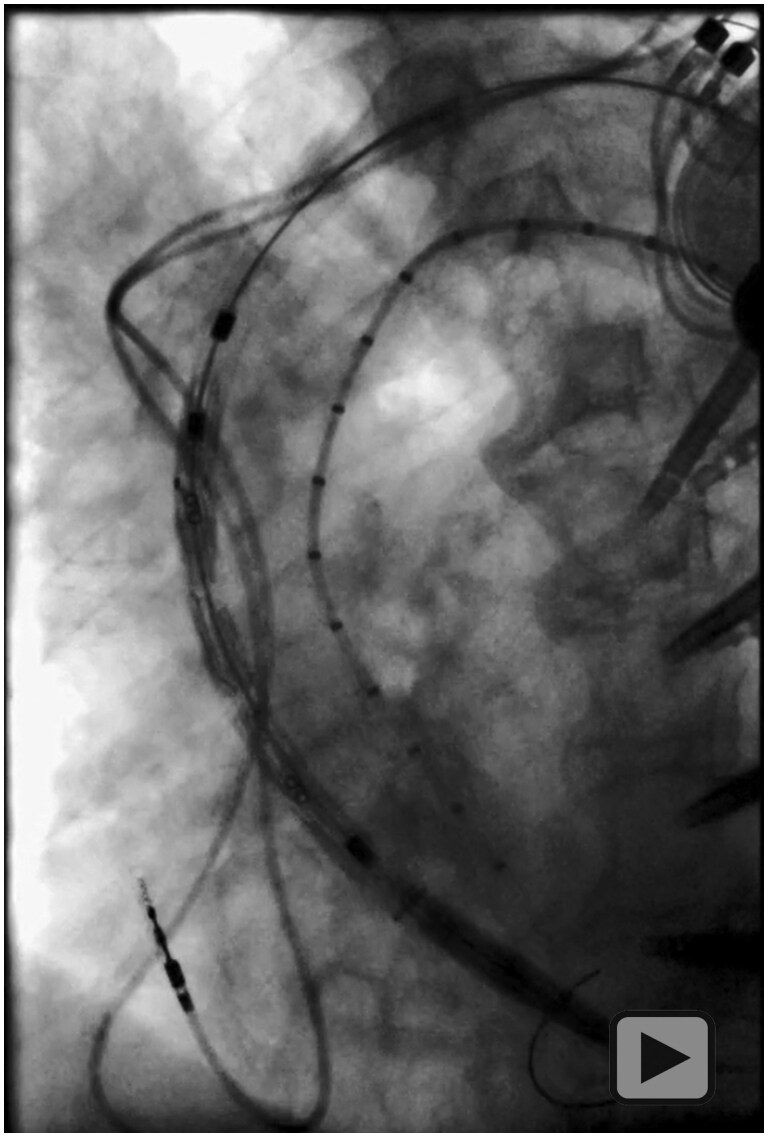
Fluoroscopic Sequence of Nexus Ascending Module (36 × 55 Mm) Deployment in Patient no. 6 for a Mycotic Pseudoaneurysm, Achieving Accurate Positioning and Complete Exclusion without Valve or Coronary Compromise.

Although 1 patient with a focal DeBakey type II dissection was successfully treated, the limited experience precludes any conclusions regarding the broader applicability of endovascular repair in type A dissection. Only one patient had a focal DeBakey type II dissection confined to the ascending aorta; therefore, no conclusions can be drawn regarding the broader applicability of endovascular repair for type A dissection.

These findings align with prior reports identifying pseudoaneurysms and localized ulcer-like lesions as ideal targets for endovascular repair due to their contained anatomy and stable landing zones^10^^,^^11^. In NAVIGATE, all such cases were treated successfully. Notably, 87.5% of patients had prior cardiac surgery, where redo sternotomy carries increased risk of graft or prosthetic injury^1^^,^^3^^,4^, making transfemoral endovascular access an attractive alternative.

Three patients (nos 5–7) had mycotic pseudoaneurysms. In 2, endovascular repair achieved early stabilization, including one (no. 7) used as a bridge to delayed homograft replacement. However, one patient (no. 6) died 4 months later from persistent infection despite initial technical success, indicating that this approach may serve as a temporizing or bridge-to-surgery strategy in selected high-risk patients but should not be considered standard treatment.

Anatomical feasibility studies further support zone 0 repair. Qiu et al. estimated that up to 52% of ascending aneurysms may be anatomically suitable for endovascular treatment with dedicated devices^9^. In NAVIGATE, procedural planning relied on ECG-gated CTA and strict sizing algorithms.

More extensive repairs for type A dissection have been reported using hybrid or branched techniques across zones 1-3^15^. Ahmed et al. reported 95.6% technical success but notable early risks (30-day mortality 9%, stroke 6%, early endoleak 18%)^16^. Similarly, the ARISE I trial demonstrated 100% technical success but 30-day mortality of 15.8% and MACCE of 26% in acute dissection^17^, highlighting the complexity of endovascular strategies in this setting.

Compared with these approaches, the NAVIGATE strategy focuses on isolated zone 0 repair without arch manipulation. Large registries have reported variable outcomes: the VQI registry described 44 ascending-only TEVAR cases with 27% perioperative mortality and ∼40% 3-year mortality^18^, while a meta-analysis by de Kort *et al.* reported 7.7% 30-day mortality and 13.1% reintervention among 259 patients^12^, with better outcomes in pseudoaneurysms.

NAVIGATE adds structured evidence by evaluating a CE-marked device specifically designed for ascending anatomy and used exclusively via transfemoral ascending-only repair. The early results—100% technical success, no mortality, and low complication rates—suggest potential advantages of dedicated devices in anatomically suitable high-risk patients. Reduced ICU and hospital stays (median 1 and 5 days) further support this approach.

These findings are consistent with the 2025 ESVS Clinical Practice Consensus Statement on ascending thoracic endovascular repair^19^, which supports the use of dedicated transfemoral devices in anatomically suitable high-risk patients following ECG-gated CTA planning and multidisciplinary evaluation.

In summary, this small multicentre case series suggests that endovascular zone 0 repair using a CE-marked dedicated endograft is technically feasible in highly selected patients. These preliminary findings require validation in larger prospective cohorts. Focal lesions such as pseudoaneurysms, penetrating ulcers, and saccular aneurysms appear to be the most suitable indications. While open surgery remains the standard for lower-risk patients, endovascular repair may represent a valuable alternative in selected high-risk or previously untreatable cases. Further studies with larger cohorts and longer follow-up are needed to confirm durability and refine patient selection.

## LIMITATIONS

This study has several limitations. Its retrospective design, small sample size, and absence of a control group limit external validity and the ability to detect rare adverse events; therefore, the absence of early mortality or major complications should not be interpreted as definitive evidence of safety. EuroSCORE II was developed for open surgery and is not validated for ascending endovascular repair, so comparisons with predicted surgical risk should be interpreted cautiously. Frailty assessment was not standardized across centers. Follow-up was limited to early outcomes, preventing conclusions regarding long-term durability, late endoleaks, graft migration, or reintervention. Patient selection was highly specific, and all procedures were performed in experienced centres, which may limit generalizability. Finally, only the ascending component of the Nexus system was evaluated, and results cannot be extrapolated to hybrid or arch-involving repairs.

## CONCLUSIONS

This small multicentre experience suggests that endovascular repair of isolated zone 0 ascending pathology using the Nexus Ascending Module is technically feasible in highly selected non-surgical candidates, with 100% technical success and no major perioperative complications. Focal lesions such as pseudoaneurysms, saccular aneurysms, and penetrating ulcers appear to be the most suitable indications. These preliminary findings require confirmation in larger prospective studies with longer follow-up.

## Data Availability

Anonymized study data may be available from the corresponding author upon reasonable request.
